# SOX9 haploinsufficiency reveals SOX9-Noggin interaction in BMP-SMAD signaling pathway in chondrogenesis

**DOI:** 10.1007/s00018-025-05622-y

**Published:** 2025-03-02

**Authors:** Tin-Yan Ha, See-Wing Chan, Zhangting Wang, Patrick Wai Nok Law, Kai-Kei Miu, Gang Lu, Wai-Yee Chan

**Affiliations:** 1 School of Biomedical Sciences, Faculty of Medicine, The Chinese University of Hong Kong, Shatin, Hong Kong SAR, China; 2 CUHK-SDU Joint Laboratory on Reproductive Genetics, School of Biomedical Sciences, The Chinese University of Hong Kong, Hong Kong SAR, China; 3 Center for Neuromusculoskeletal Restorative Medicine, Hong Kong Science Park, Shatin, New Territories, Hong Kong SAR, China; 4 Hong Kong Branch of CAS Center for Excellence in Animal Evolution and Genetics, The Chinese University of Hong Kong, New Territories, Hong Kong SAR, China; 5 Key Laboratory for Regenerative Medicine, Ministry of Education, School of Biomedical Sciences, Faculty of Medicine, The Chinese University of Hong Kong, Hong Kong SAR, China; 6 CUHK-GIBH CAS Joint Research Laboratory on Stem Cell and Regenerative Medicine, School of Biomedical Sciences, Faculty of Medicine, The Chinese University of Hong Kong, Hong Kong SAR, China

**Keywords:** Chondrogenesis, Campomelic dysplasia, Haploinsufficiency, Gene dosage, CRISPR/Cas9

## Abstract

Campomelic Dysplasia (CD) is a rare congenital disease caused by haploinsufficiency (HI) in SOX9. Patients with CD typically present with skeletal abnormalities and 75% of them have sex reversal. In this study, we use CRISPR/Cas9 to generate a human induced pluripotent stem cell (hiPSC) model from a heathy male donor, based on a previously reported SOX9 splice site mutation in a CD patients. This hiPSCs-derived chondrocytes from heterozygotes (HT) and homozygotes (HM) SOX9 mutation carriers showed significant defects in chondrogenesis. Bulk RNA profiling revealed that the BMP-SMAD signaling pathway, ribosome-related, and chromosome segregation-related gene sets were altered in the HT chondrocytes. The profile also showed significant noggin upregulation in CD chondrocytes, with ChIP-qPCR confirming that SOX9 binds to the distal regulatory element of noggin. This suggests SOX9 plays a feedback role in the BMP signaling pathway by modulating noggin expression rather than acting solely as a downstream regulator. This provides further insights into its dosage sensitivity in chondrogenesis. Overexpression of SOX9 showed promising results with improved sulfated glycosaminoglycans (GAGs) aggregation and COL2A1 expression following differentiation. We hope this finding could provide a better understanding of the dosage-dependent role of SOX9 in chondrogenesis and contribute to the development of improved therapeutic targets for CD patients.

**Supplementary Information:**

The online version contains supplementary material available at 10.1007/s00018-025-05622-y.

## Introduction

SOX9 is a transcription factor that plays a critical role in developmental events, particularly in chondrogenesis [1] and male gonadogenesis [2]. Recent studies have reported the sophisticated organizations of its cis-acting elements, which spans 0.5 Mb downstream and 1.9 Mb upstream, regulating gene expression across 13 tissues types, such as rib, prechondrocytes and proliferating chondrocytes [1]. Campomelic Dysplasia (CD) is a complicated developmental disease caused by a loss-of-function allele in SOX9. Symptoms include clubfeet and bending of long bones. In addition to skeletal abnormalities, XY sex reversal and ambiguous genitalia affected 75% of the patients [3, 4]. The radiological assessments revealed global skeletal abnormalities in CD patients, including cartilage related features such as flat nasal bridge, club foot, tracheomalacia; mixture of cartilage and bone complications include bowed bones, missing a pair of ribs, non-mineralized thoracic pedicles, small thorax, etc.; Few patients with CD survive into adolescence, most of them perished shortly after birth due to respiratory failure [5, 6]. The estimated prevalence of CD ranges from 1 in 40,000 to 80,000, with additional cases being reported [7].

As the skeletal system develops, mesenchymal stem cells undergo chondrogenesis and become chondrogenic progenitor cells, which proliferate and mature into chondrocytes. Depending on the niche, these chondrocytes can form different types of cartilage, such as hyaline and articular cartilage by homeostasis, or undergo hypertrophy to orchestrate endochondral ossification.

SOX9 is crucial in chondrogenesis and chondrocyte homeostasis. Its expression is necessary in the early mesenchyme to form pre-cartilaginous condensations [8, 9]. SOX9 drives the transcriptions of cartilage matrix genes, such as ACAN, COL2A1, COL9A1, and COL11A2, thereby promoting chondrogenesis [10–13]. The initiation of SOX9 expression during embryogenesis was induced by Notch signaling to drive chondrogenic differentiation in embryoid bodies [14]. N-cadherin [15] and BMP signaling pathways [16–18] also upregulate SOX9 for the initiation of chondrogenesis. SOX9 acts downstream of FGF2-induced proliferation but does not response to iGF1, BMP2, or BMP7-induced proliferation in adult articular chondrocytes, suggesting its role in chondrocyte proliferation is in a context-dependent manner [19]. In cartilage homeostasis, SOX9 directly activates miR-140 expression, which is essential in cartilage homeostasis and development [20]. TGF-β phosphorylates and stabilizes SOX9 protein via p38 and SMAD to facilitate chondrocyte homeostasis [21]. As chondrocytes reach hypertrophy, SOX9 expression is vital to sustain chondrocyte survival, directing hypertrophy, and preventing premature endochondral ossification [22–24]. During hypertrophic differentiation, SOX9 decreases to alleviate the suppression of osteogenic genes such as MMP13, COL1A1, and IBSP [25]. In summary, SOX9 acts as the downstream of various signaling pathways and promotes chondrogenic gene transcriptions, orchestrating the skeletal development from chondrogenesis to endochondral ossification. The skeletal abnormalities resulting from a single copy loss of SOX9 allele in CD patients highlight the critical role of SOX9’s dosage in proper formation of cartilage and bone.

In addition to its role in developmental processes, SOX9 also engages in cartilage homeostasis and regeneration. Downregulated SOX9 has been reported in osteoarthritic articular cartilage [26, 27], while osteosarcoma [28] and chondrosarcoma [29] exhibit upregulation of SOX9 together with proliferative chondrocytes. These findings suggest that abnormal SOX9 expression can have detrimental effects in various disease contexts. Nevertheless, the investigation of SOX9 HI’s effects is hampered by the lack of human cell models.

The powerful genome editing technology CRISPR/Cas9 has been widely used to model various hereditary disorders [30]. Base editors, built on the CRISPR/Cas9 system, enable editing single base pair without inducing DNA double strand breaks, making genome editing more efficient and adaptable [31]. Induced pluripotent stem cell (hiPSCs) are promising sources for disease modeling due to their ability to differentiate into various cell types. This is particularly beneficial for studying the pathophysiology and underlying molecular pathways affected by disease-causing mutations. The limited availability of patient cells for rare genetic disorder could be addressed by combining CRISPR/Cas9 genome editing and hiPSCs [32]. Moreover, isogenic clones have the edge over patient cell lines for minimizing the biological variance caused by genetic background among individuals.

In this study, using CRISPR/Cas9 genome editing technology and hiPSCs from a healthy person, we present the first SOX9 HI model produced in hiPSCs by replicating a disease-causing single-point mutation in the SOX9 intron 2 splice site from a CD patient [3]. Transcriptome analysis and rescue models have also been performed and established. With the discovery of the SOX9-noggin feedback loop in BMP signaling by ChIP-qPCR and successfully restoring HI mutant chondrocytes via SOX9 overexpressing, we hope to provide insights to develop novel therapeutic strategies for CD patients.

## Materials and methods

### Maintenance of hiPSCs

hiPSCs were derived from a male healthy donor dermal fibroblast in our lab and characterized in the previous paper [33]. hiPSCs were seeded on Matrigel® Matrix (Corning®, 354277) coated 6-well plates. They were cultured in mTeSR™1 (STEMCELL Technologies, 85850) medium and the medium was changed daily.

Cells were passaged using Dispase (STEMCELL Technologies, 85850) when 80% of confluence was reached. In brief, cells were washed with PBS once and incubated with Dispase at 37 °C for 5 min. Dispase was discarded. Cells were washed with PBS three times gently. Cells was lifted in the mTeSR™1 medium by cell scraper and transferred to a new coated well. The split ratio was 1:4 to 1:10 depending on the confluence.

### Cloning of SOX9 sgRNA

SOX9 sgRNA target sequence was designed based on MIT CRISPR Tool (Supp. Tables). The target sequence was cloned into MLM127 (Addgene#43860) for sgRNA expression. sgRNA oligonucleotides were annealed and phosphorylated in T4 Polynucleotide Kinase (T4 PNK) (NEB, M0201S) and T4 DNA Ligase Reaction Buffer (NEB, B0202S). The reaction mix was incubated in a thermocycler at 37 °C for 30 min, 95 °C for 5 min, then ramped down to 25 °C at 5 °C per min according to Zhang’s protocol [34]. The phosphorylated and annealed sgRNA oligos were diluted 200-fold with ddH2O. The MLM127 plasmid was cut with BsmBI (NEB, R0580) and the sgRNA oligos were ligated in T4 DNA Ligase Reaction Buffer with T4 DNA Ligase (NEB, M0202S). The reaction mix was incubated overnight at 16 °C. The ligation products were then transformed using TaKaRa E. coli DH5 α Competent Cells (TaKaRa, 9057). In brief, 2 µl of ligation mix was added to 50 µl of DH5 α. The competent cells were incubated on ice for 20 min, at 42 °C for 45 s, and then 2 min on ice for heat shock. 250 µl of SOC medium was added to the competent cells and were incubated at 37 °C with shaking for 1 h. The competent cells were spread on ampicillin LB agar plates and incubated at 37 °C for 16 h. The next day, single clones were picked and amplified in 3 mL of LB broth supplemented with 1 µg/mL of ampicillin (Sigma, A0116) for 16 h. The plasmids were extracted with Mini Plus Plasmid DNA Extraction System (VIOGENE, GF2002) and were sequenced to ensure that cloning was successful.

### Electroporation of CRISPR/Cas9 components in hiPSCs

Single cell suspension of hiPSCs in mTeSR™1 medium supplemented with 1 µM Y-27632 2HCl (aka. ROCK inhibitor) (Selleckchem, S1049) and was obtained by incubating in ACCUTASE™ (STEMCELL Technologies, 07920) for 5 min at 37°C, pipetting up and down gently to homogenize, and centrifugation at 100 g for 5 min. hiPSCs were then electroporated with the third generation base editor, BE3 (Addgene#73021) [31], sgRNA (MLM127 cloned with the SOX9i sgRNA), and GFP (Provided by Clontech; Addgene#32538) in the ratio of 8:3:1. A total of 5 µg of DNA was electroporated with Human Stem Cell NucleofectorTM Kit 1 (Lonza, VPH-5012) according to manufacturer’s instructions. After electroporation, cells were plated onto newly coated Matrigel plates supplemented with CloneR™ (STEMCELL Technologies, 05888) in mTeSR™1 medium. mTeSR™1 medium supplemented with CloneR™ was changed daily. CloneR™ was lifted after colonies were observed.

### Fluorescence-activated cell sorting (FACS) and single clone selection

hiPSCs were dispersed into a single-cell suspension by using ACCUTASE™. Cells were washed in PBS and were concentrated by centrifugation at 200 g for 3 min. PBS was discarded and the cell pellet was resuspended in 1 mL of BD stain buffer (BD, 554656). The cells were transferred to a flow tube with cell strainer cap to obtain single cell suspension. GFP-positive cells were sorted and collected by BD FACS Aria II Cell sorter. Sorted cells were plated on newly coated Matrigel 6-well plates supplemented with CloneR™ in mTeSR™1 medium with 1000, 2000, and 5000 cells seeded per well. mTeSR™1 medium supplemented with CloneR™ was changed daily. Single colonies were picked and transferred to newly-coated Matrigel 24-well plates. CloneR™ was lifted after single colonies were transferred to 24-well plates.

### Genomic DNA extraction and PCR

Genomic DNA of each clone was extracted by PureLink TM Genomic DNA Mini Kit (Thermo Scientific™, K182001) according to manufacturer’s instructions. 200 ng of gDNA was used for amplifying the targeted SOX9 fragment by PCR with Phusion® High-Fidelity DNA Polymerase (NEB, M0531S). The PCR products were sent to Sanger sequencing for detection of mutagenesis. Primer sequence is provided in Supp. Tables.

### Chondrocyte differentiation from hiPSCs

The chondrocyte differentiation was based on the protocol established by Oldershaw et al. [35]. In brief, hiPSCs were seeded as single cells at 40–50% confluence in mTeSR™1 medium before the first day of differentiation (around 9 × 10^5^ cells per 6-well plate). The basal medium for hiPSC-derived chondrocytes includes DMEM/F-12 (Gibco™, 11320033) medium, 2 mM GlutaMAX™ Supplement (Gibco™, 35050061), 1% 100X Insulin-Transferrin-Selenium (Gibco™, 41400045), 1% MEM Non-Essential Amino Acids Solution (100X) (Gibco™, 11140050), 2% B-27™ Supplement (Gibco™, 17504044) and 90 µM 2-mercaptolethanol (Gibco™, 21985023). 7 chemicals, Recombinant Human Wnt-3a Protein (R&D Systems, 5036-WN-010), Recombinant Human/Murine/Rat Activin A (PEPROTECH, 120-14P), bFGF (Thermo Scientific™, PHG6015), BMP4 (Invitrogen, PHC9533), Follistatin 300 human (Sigma, F1175-25UG), GDF-5 human (Sigma, SRP4667-50UG), and Recombinant Human NT-4 Protein (R&D Systems, 268-N4-005) were used. Differentiating cells were passaged with Trypsin–EDTA whenever 80–90% confluence was reached until Day 14.

According to the chondrocyte differentiation matrix developed by Oldershaw et al. [35], 25 ng/mL WNT3A was added to the basal medium from day 1 to 3. 50 ng/mL, 25 ng/mL, and 10 ng/mL of Activin-A were added from day 1 to 3, respectively. 20 ng/mL of FGF2 was added from day 2 to 13. 40 ng/mL of BMP4 was added from day 3 to 8, and decreased to 20 ng/mL from day 9 to 10. 100 ng/mL Follistatin 300 was added from day 4 to 7. 20 ng/mL GDF5 was added day 9 to 10, and the concentration was increased to 40 ng/mL per day from day 11 to 13. 2 ng/mL NT4 was added from day 4 to 14.

### RNA extraction and RT-qPCR

Cells collected were dissolved in 0.5 mL of QIAzol Lysis Reagent (QIAGEN, 79306). 100 µl of chloroform was added and the lysate was vortexed and centrifuged for 12 min at 4 °C at maximum speed. The uppermost supernatant was extracted and mixed with absolute ethanol in a 1:1 volume ratio. Direct-zol RNA Miniprep Kit (ZYMO RESEARCH, R2052) was used for subsequent steps following manufacturer’s instructions. Reverse transcription is performed using PrimeScript™ RT Master Mix (Perfect Real Time) (TaKaRa, RR036B) following manufacturer’s instructions. TBGreen (TakaRa, RR420) was used for RT-qPCR on 384 well-plates. Gene expression was normalized with GAPDH and expressed in the form of mean ± sem. SOX9 qPCR primers were designed to target exon 2 to exon 3 region only to exclude the SOX9 mutant mRNA. Primer sequences are provided in Supp. Tables.

### Transcript PCR

RNA was extracted and reversely transcribed as per the similar method mentioned in the RNA extraction and RT-qPCR section. cDNA was diluted tenfold and measured for concentration. 200 ng of DNA samples were used in a 20 µL system by GoTaq® Master Mixes (Promega, M7122). The PCR product was separated by 2% TAE agarose gel (Bio-rad, 1613102) at 100 V for 30 min. Primer sequences are provided in Supp. Tables.

### Western blot

Cell pellets were lysed and collected in RIPA Lysis and Extraction Buffer (Thermo Scientific™, 89900) on ice. The concentration of protein lysate was estimated accordingly by Pierce™ BCA Protein Assay Kit (Thermo Scientific™, 23225) according to manufacturer’s instructions. The denatured samples were prepared by adding 5 × sample buffer containing β-mercaptoethanol and heated for 5 min at 99 °C. 20 µg of the samples were loaded to 10% SDS-PAGE separation gel. The samples were transferred to a PVDF membrane using the Mixed-Range MW program in Power Blotter System (Invitrogen™, PB0012). The membrane was blocked for 1 h at room temperature with blocking solution containing Blotting-Grade Blocker (Bio-Rad, 170–6404) in PBS-T. SOX9 and GAPDH (Cell Signaling TECHNOLOGY, 2118S) primary antibodies were diluted at ratio of 1:1000 and 1:2000 in blocking solution, respectively, and incubated at 4°C overnight and were washed with PBS-T for 3 times with 15 min each. On the next day, the membrane was washed with PBS-T for 3 times with 15 min each. Anti-rabbit IgG (Cell Signaling TECHNOLOGY, 7074S) was diluted at 1:5000 and was incubated at room temperature for 1 h. Bands were visualized after being washed by PBS-T for 3 times by Pierce™ ECL Western Blotting Substrate (Thermo Scientific™, 32109) according to manufacturer’s instructions.

### Immunofluorescence staining

Cells were fixed with 4% paraformaldehyde (PFA) (Sigma, F8775) for 20 min. For staining of intracellular markers, cells were permeabilized with 0.1% Triton® X-100 (Affymetrix, 22686) in blocking solution for 45 min at room temperature. The blocking solution contained 1% Bovine Serum Albumin (BSA) (Sigma, A4161) in PBS with 4% Normal Goat Serum (Abcam, ab7481). SOX9 (Millipore, AB5535) and COL2A1 (SANTA CRUZ BIOTECHNOLOGY, sc-518017) primary antibodies were diluted at the ratio of 1: 200 and 1: 100 respectively in blocking solution and incubated overnight at 4 °C with shaking in the dark. After washing with PBS, Alexa-555-conjugated goat anti-rabbit (Invitrogen™, A27034) and Alexa-488-conjugated goat anti-mouse (Invitrogen™, A28180) were used and diluted at the ratio of 1:1000 in blocking solution. Secondary antibodies were incubated for 2 h at room temperature in the dark with shaking. Cells were washed with PBS. NucBlue™ Fixed Cell ReadyProbes™ Reagent (DAPI) (Invitrogen™, R37606) was diluted in PBS and washed together for 5 min with PBS for nuclei visualization. Cells were preserved with Prolong Gold antifade (Thermo Scientific™, P10144) with cover glass and were observed under confocal microscope. Cell fluorescence was quantified using ImageJ [36].

### Alcian blue staining

Cells were washed with PBS three times and fixed with 4% PFA for 20 min. Cells were stained with Alcian Blue Solution (Sigma-Aldrich, B8438-500ML) for 15 min, then washed with 70% ethanol and ddH_2_O. Cells were covered with PBS to avoid drying out and were observed under microscope.

### Transcriptome analysis

RNA samples were sent to Novogene Company for bulk RNA-sequencing. Gene raw count was obtained by using STAR-rsem pipeline using hg38 reference [37, 38]. EdgeR R package [39] was used for differential expression analysis. Genes with an adjusted P value ≤ 0.05 and with a net log fold change ≥ 1.5 were considered to be differentially expressed. Gene Ontology (GO) enrichment analysis of differentially expressed genes and GSEA analysis were performed by the clusterProfiler R package [40]. GO terms and GSEA terms with adjusted P values less than 0.05 were considered significantly enriched.

### Prediction of binding sites

The SOX9 motif file is downloaded from the Motif Databases in HOMER. The motif file was compared against the hg38 genome by the HOMER software [41] to perform the analysis using the scanMotifGenomeWide.pl function.

### Chip-qPCR

Chip-qPCR samples were collected directly from 6-well plate culture on Day 14 of chondrocyte differentiation by scraping. Methods from sample collection to DNA clean-up were followed by the Pierce™ Magnetic ChIP Kit instructions (Thermo Scientific™, 26157), except the chromatin was fragmented by sonication instead of MNase digestion. In brief, cells were fixed with PFA for 10 min and followed by quenching with glycine for 5 min at room temperature. The cell pellets were washed twice by cold PBS. Cells were scraped in PBS with proteinase/phosphatase inhibitor provided by the kit and centrifuged at 3000 × *g* for 5 min. After incubating in the membrane extraction buffer with inhibitor on ice for 10 min, nuclei were centrifuged and resuspended in IP Dilution Buffer with inhibitor for sonication. Sonication was performed on a focused ultrasonicator (Covaris, ME220) with two repeats of 67 peak power, 20% duty factor, and 1000 cycles per burst at 6 °C for 35 s. Chromatins were collected by centrifugation at 9000 *g* for 5 min. Input samples were collected at this stage, and the rest of the samples were proceeded to primary antibody incubation overnight at 4 °C with shaking. The samples were bound to protein A/G magnetic beads, washed, and eluted. The eluted samples and input samples then underwent crosslink reversal with proteinase K treatment at 65 °C for 1.5 h. DNA was purified by using the column and wash buffers provided by the kit. Primers were designed using the Primer-BLAST website with the predicted binding sites sequence retrieved from the UCSC Genome Browser. qPCR was performed with PowerTrack™ SYBR Green Master Mix (Applied Biosystems™, A46012). Samples were normalized with respective input samples and IgG samples.

### Packaging of lentiviruses

Two viruses were prepared, including FUW-TetO-Sox9 (Addgene#41080) and FUW-m2rtTA (plasmid #20342). Healthy 293T cells were transfected with jetPRIME® (Polyplus, 114-15) for each virus, together with 2 packaging plasmids pMD2.G (Addgene, plasmid #12259) and psPAX2 (Addgene, plasmid #12260), following manufacturer’s instructions. Medium was collected and filtered by a 0.45 µm filter. PEG-it™ Virus Precipitation Solution (5 ×) (System Bioscience, LV825A-1) was added to precipitate the viral particles. The viruses resuspended in PBS and were stored in a − 80 °C freezer for long-term storage.

### Induction of SOX9 expression by doxycycline during chondrocyte differentiation

hiPSCs were differentiated to chondrocytes according to the methods described previously. On Day 9 of chondrocyte differentiation, 10 µl lentivirus of FUW-TetO-Sox9 (Addgene#41080) was added together with 20 µl of FUW-m2rtTA (plasmid #20342) lentivirus in the supplement of 8 µg/mL polybrene. 48 h later, 1 µg/mL of doxycycline (dox) (Clontech, NC0424034) was added to induce the expression of SOX9. Dox was replaced daily, and cells were collected on Day 14 for subsequent studies.

## Results

### Generation of CRISPR/Cas9-mediated SOX9 HI hiPSC model

**Fig. 1:**
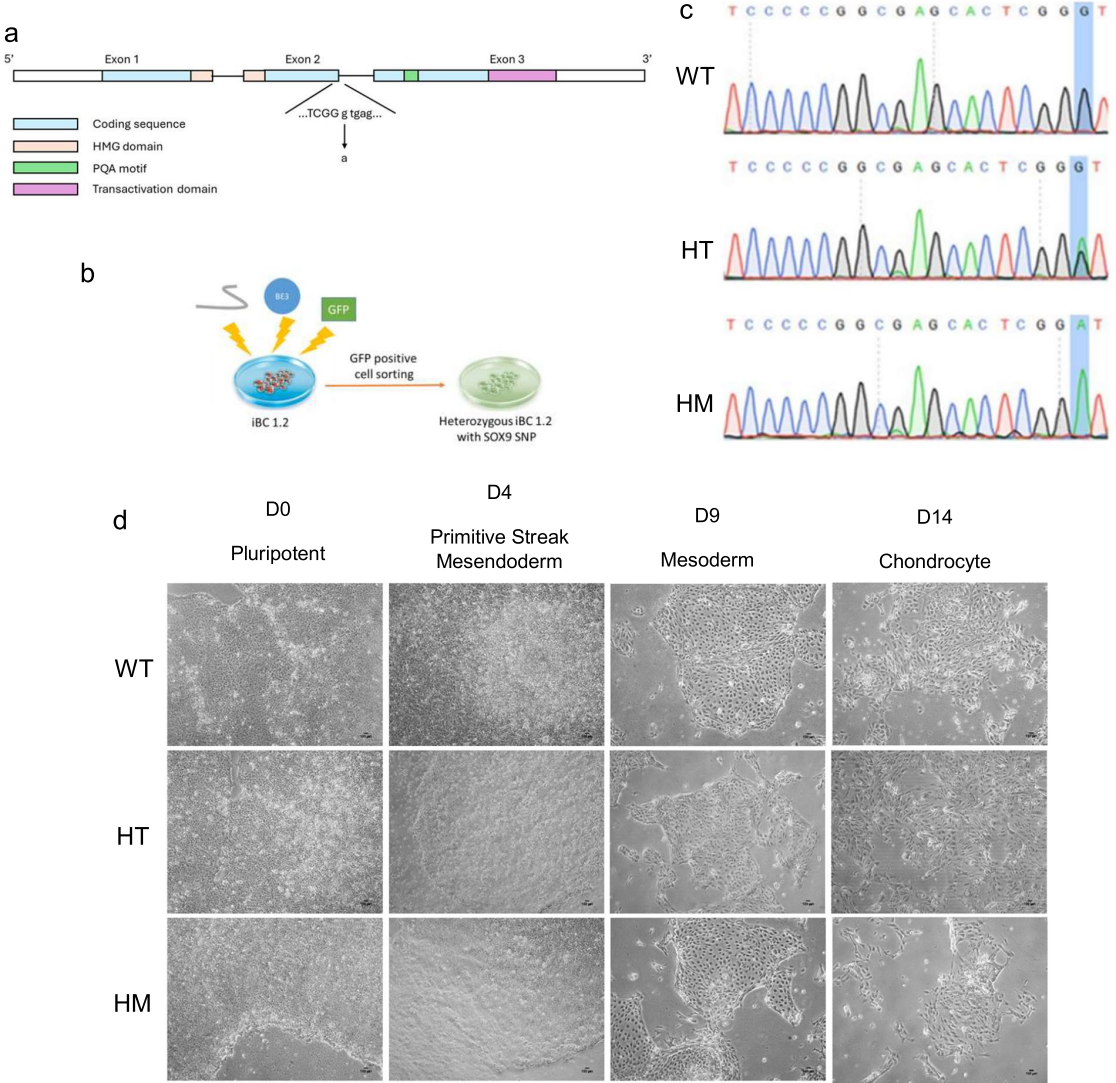
SOX9 haploinsufficient mutagenesis and validation. **a** Illustration of the SOX9 HI single-point mutation at intron 2 with lower case representing intron sequence. **b** SOX9 mutagenesis by CRISPR/Cas9 in hiPSCs. hiPSCs were electroporated with BE3, sgRNA, and a GFP plasmid. **c** Sanger sequencing of wild-type (WT) and SOX9-edited (HT and HM) hiPSCs. **d** Morphology of wild-type and SOX9 edited (HT and HM) hiPSCs during chondrogenic differentiation

We established our SOX9 HI model in a hiPSC cell line named iBC1.2 which is previously established in our lab [33]. We discovered a G to A single-point mutation at the SOX9 intron 2 splice sites (Variant Identifiers: NM_000346.4(SOX9):c.685 + 1G > A) through a search of the medical literature [3] (Fig. 1a). By electroporation of the CRISPR/Cas9 components, including sgRNA, BE3, and a GFP plasmid, we attempted to recreate this mutation in our hiPSC line.

GFP-positive cells were sorted and cultivated into single clones after three days (Fig. 1b). 25 single hiPSC clones’ PCR products were sent for Sanger sequencing to check for the SOX9 HI mutation. Two clones, representing heterozygous (HT) and homozygous (HM) single point mutations, were selected. HT represented the expected SOX9 mutation, the same as the CD patient, with a modest green peak suggesting an A on one allele and a G on the other. A green signal in the Sanger sequencing of HM indicates that both alleles have undergone a G to A mutation (Fig. 1c). These two clones were selected to establish an isogenic basis for studying SOX9 gene dosage on development, TA cloning was also performed to confirm the genotype of individual alleles for each cell line (Supp. Tables).

### Characterizations of hiPSC-derived chondrocytes showed weakened chondrocytes markers in SOX9 HI mutants

**Fig. 2:**
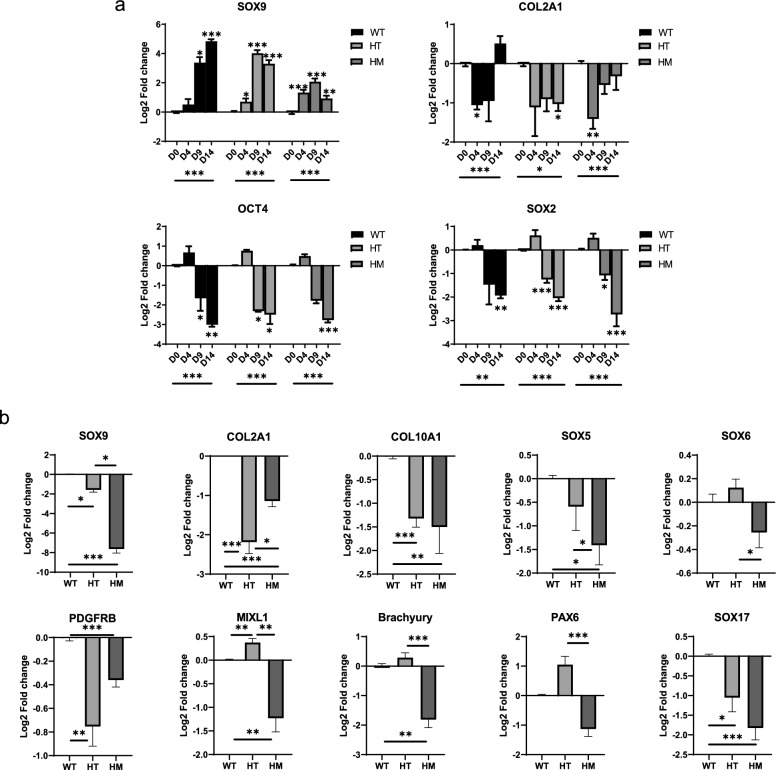
Characterization of gene expression of hiPSC-derived chondrocytes. **a** Gene expression of chondrogenic markers (SOX9 and COL2A1) and pluripotent markers (OCT4 and SOX2) along different stages of chondrogenesis in different phenotypes (WT, HT, and HM). **b** Gene expression of chondrogenic markers (SOX9, COL2A1, and COL10A1), chondrogenic precursor markers (SOX5, SOX6, and PDGFRB), germ layer markers (Brachyury, PAX6, and SOX17), and primitive streak mesendoderm markers (MIXL1) in different phenotypes on D14. (N = 3, *P < 0.05, **P < 0.01, ***P < 0.001)

After successful generation of the SOX9 HI model, these hiPSCs with three genotypes (WT, HT and HM) were differentiated into chondrocytes according to a well-established protocol [35]. Briefly, cells underwent three stages: primitive streak mesendoderm (D4), mesoderm (D9) and chondrocyte (D14). Figure 1d illustrates the morphology of all 3 genotypes along differentiation at different stages. Although there was no obvious morphological difference among the three genotypes, SOX9 expression among them was distinctive (Fig. 2a). During differentiation, SOX9 expression increased by about 5 folds in WT from D0 to D14. In HT group, there was an increase of SOX9 by about 10 folds from D0 to D9, while on D14, expression of SOX9 decreased. In HT and HM groups, SOX9 expression increased from D0 to D9 and decreased on D14. To compare the difference among three genotypes, we examined the expression of genes from different categories including chondrocyte markers, germ layer markers, and primitive streak mesendoderm markers on D14 (Fig. 2b). SOX9, COL2A1, and COL10A1 were selected as the chondrocyte markers. The expression level of SOX9 was very distinctive among different genotypes on D14. When compared to WT, HT and HM chondrocytes showed a significant decrease in COL2A1 and COL10A1 expression. PDGFRB, as one of the chondrogenic precursor markers, was significantly downregulated in both HT and HM. For SOX5 and SOX6, there were no significant differences between HT and WT. Similar trends were found in the mesoderm marker Brachyury. The expression of mesendoderm markers MIXL1 showed a slight increase in HT, while HM showed a drop when compared to WT. For the endoderm marker SOX17, the expression in both HT and HM decreased significantly.

**Fig. 3:**
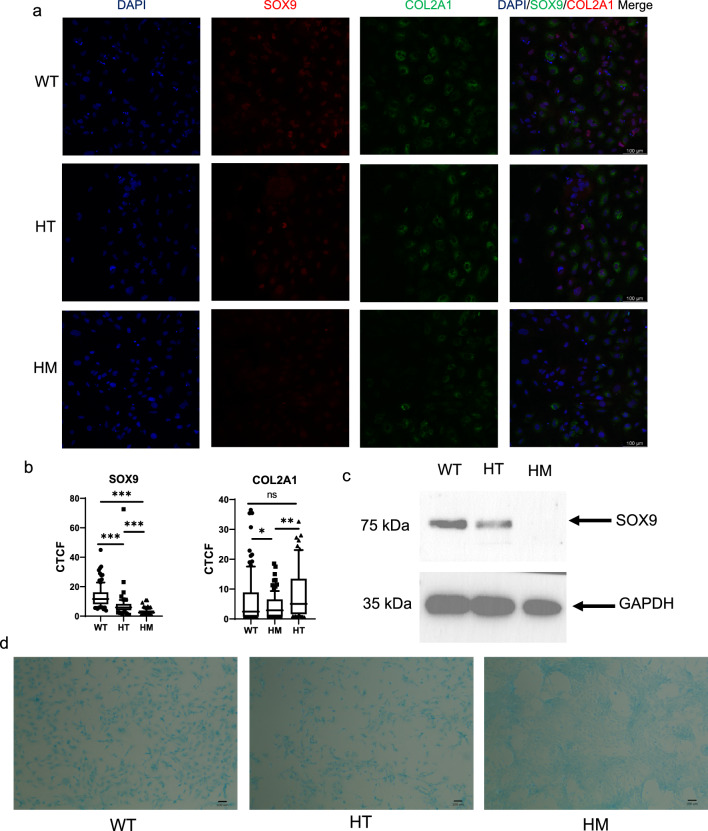
Characterization of SOX9 HI hiPSC-derived chondrocytes. **a** Immunofluorescence staining of Day 14 hiPSC derived chondrocytes. **b** Quantification of COL2A1 and SOX9 Corrected Total Cell Fluorescence (CTCF) of **a**. **c** Western blot analysis of hiPSC-derived chondrocytes. Full-length blots/gels are presented in Supp. Fig. 1. **d** Alcian blue staining of the hiPSC-derived chondrocytes on Day 14

Apart from gene expressions, the protein expressions of D14 chondrocytes were examined by immunofluorescence (IF) staining and Western blot. Following a similar trend in the gene expression results, the protein expression of SOX9 on D14 between the 3 genotypes was also distinctive in IF and Western blot (Fig. 3a and c). In the IF staining, the expression level of SOX9 in HM and HT was significantly lower than that of WT, and the COL2A1 protein level in HT was less than that of WT (Fig. 3b). For WT IF staining, most of the SOX9 expressions were co-localized with the DAPI signal. However, HM mutSOX9 was localized in cytoplasm, and a similar phenomenon also happened in part of the HT IF staining.

As a functionality test of chondrocyte, we examined the ability to accumulate GAGs in different genotypes by alcian blue staining on D14 (Fig. 3d). The amount of GAGs was reduced in HT and HM genotypes compared to WT hiPSCs-derived chondrocytes, which indicates that the ability to accumulate GAGs was weakened upon decrease in SOX9 gene dosage.

### HT chondrocytes showed no evidence in nonsense-medicated decay response

While there are two SOX9 mRNA isoforms in frogs [42], according to the gene report in NCBI, human SOX9 has never been reported with any isoforms. As our model has a point mutation at the splice site between exon 2 and exon 3, it is crucial to examine the nonsense-mediated decay (NMD) pathway to see if the mutated SOX9 mRNA was degraded.

**Fig. 4:**
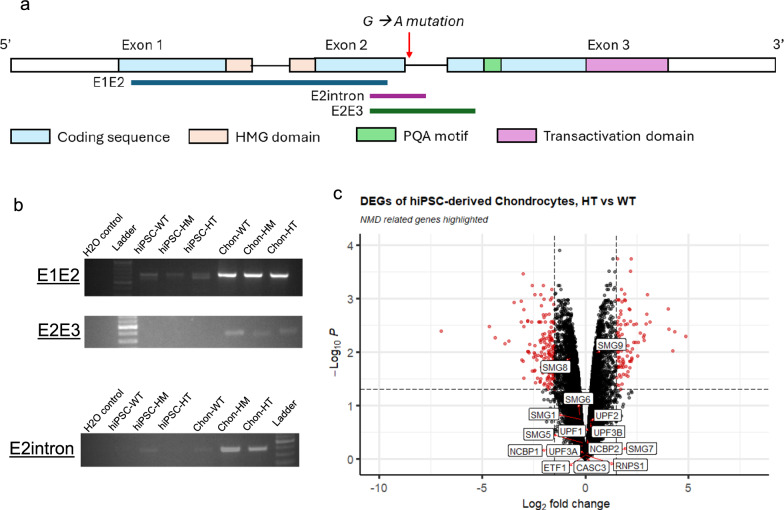
Nonsense-mediated decay validation. **a** Illustration of SOX9 mRNA structure and the target RT-PCR product. **b** RT-PCR result of SOX9 mRNA in WT, HM, and HT before and after chondrocytes differentiation. Full-length gels are presented in Supp. Fig. 2. **c** Volcano plot from RNA seq showing the NMD factors with DEG presented in red

Primers targeting exon 1 to exon 2 (E1E2), exon 2 to intron 2 (E2intron), and exon 2 to exon 3 (E2E3) of the SOX9 mRNA were designed as illustrated in Fig. 4a. The RT-PCR result showed HT and HM expressed the SOX9 mRNA. It also suggested a more abundant expression of SOX9 mRNA in chondrocytes than that in hiPSC. In the E1E2 fragment, there is no obvious difference between WT, HT, and HM. However, in the E2intron, both HT and HM chondrocytes showed evident intron retention, which is expected from the mutation at the exon 2 splice site. For E2E3, the HM chondrocytes band was weaker than both the WT and HT counterparts, suggesting less proportion of HM SOX9 mRNA covered the exon 3 region. The above results suggested that after the mutated splice site, there was intron retention and fewer transcription events could reach exon 3 in HT and HM chondrocytes.

RNA seq transcriptome profiling allowed us to see if any NMD-related genes were significantly altered in the HT chondrocytes. As shown in the volcano plot in Fig. 4c, all the NMD factors were in the non-significant region in the differential analysis comparing HT chondrocytes to WT, suggesting that despite the expression of mutSOX9, NMD pathway in the HT chondrocytes showed no signs in alteration when compared to WT chondrocytes.

### Transcriptome analysis of SOX9 HI hiPSC-derived chondrocytes

**Fig. 5:**
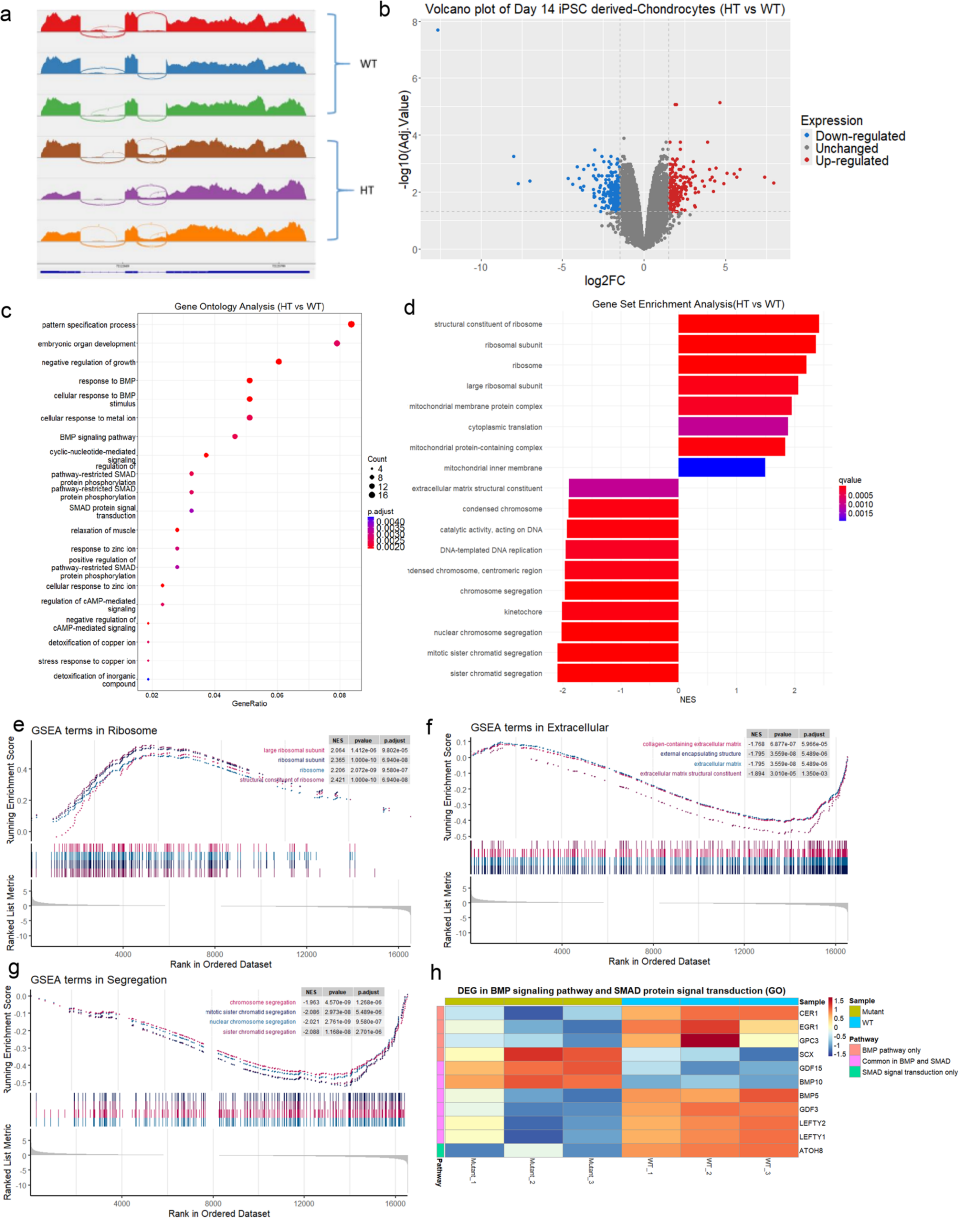
RNA-seq analysis of SOX9 HT hiPSC-derived chondrocytes. **a** Screen capture of the SOX9 gene fragments by Integrative Genomics Viewer (IGV) on WT and HT hiPSCs-derived chondrocytes. **b** Volcano plot of SOX9 WT and SOX9 HT chondrocytes with estimated 400 genes differentially expressed. **c** Dot plot of the Gene ontology result. **d** Bar plot of the GSEA result showing the normalized enrichment score (NES). GSEA plots showing the enriched gene sets related to ribosome **e**, extracellular matrix (**f**), and chromosome segregation (**g**). **h** Heatmap of the DEGs in the BMP and SMAD signaling pathway

Bulk RNA sequencing was performed to clarify the potential pathways and mechanisms impacted by the SOX9 gene dosage in chondrocytes. The SOX9 splicing was accessed using Integrative Genomic Viewer, and the readings from HT hiPSC-derived chondrocytes were shown to have intron 2 retention (Fig. 5a).

About 400 genes were identified as differentially expressed in the RNA-seq statistical analysis, as shown in the volcano plot (Fig. 5b). Several biological processes related to pattern specification and the BMP-SMAD signaling pathway were enriched in GO analysis (Fig. 5c). In the Gene Set Enrichment Analysis (GSEA) analysis, processes related to ribosome, chromosome segregation, and extracellular matrix (ECM) were enriched (Fig. 5d). Ribosome related processes were upregulated (Fig. 5e) whereas ECM and chromosome segregation were downregulated in HT chondrocytes when compared to WT (Fig. 5f–g). A summary of differentially expressed genes (DEGs) involved in the BMP and SMAD signaling pathways is shown in Fig. 5h. Full tables of DEGs, enriched GO and Gene Set terms are documented in Supp. Tables.

**Fig. 6:**
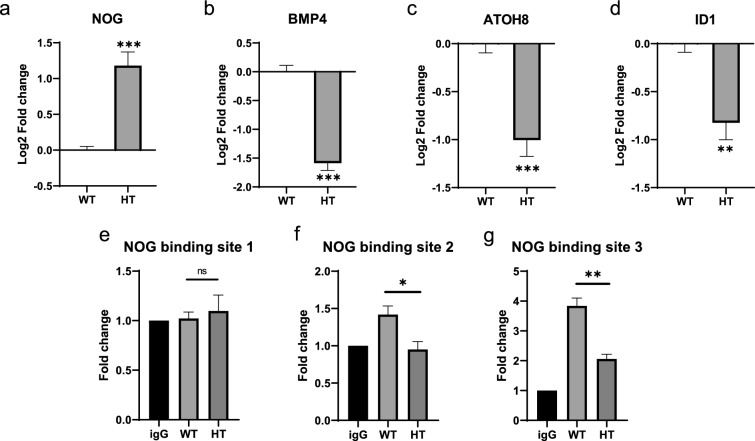
RNA-seq validation on BMP signaling pathway of SOX9 HT hiPSC-derived chondrocytes. Gene expression of BMP antagonist, NOG (**a**); BMP ligand, BMP4 (**b**); BMP modulator, ATOH8 (**c**); BMP target, ID1 (**d**). Chip-qPCR on SOX9 predicted binding sites at NOG regions (**e–g**)

To validate the result, gene expressions of NOG, BMP4, ATOH8, and ID1 were examined by RT-qPCR. Expressions of the BMP4, ATOH8, and ID1 involved in the BMP pathway were all downregulated in HT chondrocytes significantly (Fig. 6b–d), meaning the pathway might be suppressed when only half of the SOX9 dosage was available. At the same time, the inhibitor of the BMP pathway, NOG, was upregulated in the HT mutant (Fig. 6a). We performed a ChIP-qPCR on the WT and HT chondrocytes to examine if SOX9 could bind to NOG-regulating regions with the aid of HOMER software to screen the possible binding sites (Supp. Table). We found that SOX9 could minutely bind to the second predicted site at 3k downstream to NOG transcription start site (TSS) while there was no enrichment in HT when compared to the IgG control (Fig. 6f). For the third predicted binding site located at 9 kb upstream of the NOG TSS, it showed significant enrichment in both WT and HT chondrocytes, with WT showing higher binding than HT significantly (Fig. 6g).

**Fig. 7:**
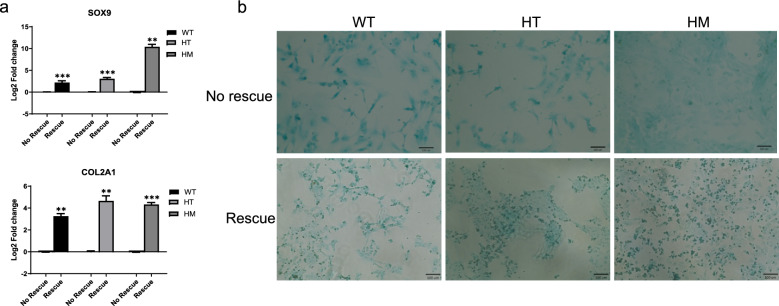
Rescuing SOX9 HI mutants by SOX9 overexpression. **a** Expression of SOX9 and COL2A1 after overexpression of TetOn SOX9 on D14 hiPSC-derived chondrocytes compared with the non-treated group of each genotype. (N = 3, *P < 0.05, **P < 0.01, ***P < 0.001) **b** Alcian blue staining on D14 of hiPSC-derived chondrocytes in the No Rescue (upper panel) and Rescue (lower panel) groups

### Rescue SOX9 HT mutants by overexpression of SOX9

To rescue SOX9 HT mutants in hiPSC-derived chondrocytes, lentiviruses of FUW-TetO-Sox9 and FUW-m2rtTA from Rudolf Jaenisch [43] were infected on D9 of differentiation, where the cells were in mesoderm stage and started to express SOX9 under normal conditions. Dox was added to induce SOX9 expression 48 h later and cells were collected on D14 together with non-treated controls (No Rescue). On D14, SOX9 expression of the Rescue group increased when compared to the No Rescue group, indicating SOX9 was successfully overexpressed upon dox induction. For another chondrocyte marker, COL2A1, the expression level in the rescue treatment group of all 3 genotypes also increased. These results indicated that with rescue treatment, not only SOX9 but also the gene expression level of COL2A1 could be rescued (Fig. 7a). Alcian blue staining also showed increased blue colour intensity in the rescued D14 cells, indicating the accumulation of GAGs in HT and HM chondrocytes was improved upon the induction of TetOn SOX9 (Fig. 7b).

## Discussion

The precise number of HI genes in the human genome is still unclear, but it is estimated that approximately 10% of genes could not tolerate heterozygous changes. HI has long been known to cause various diseases, including developmental disorders. The association between dosage sensitivity and HI highlights the non-linear relationship observed between genotype and phenotype [44, 45]. As a SOX transcription factor, SOX9 is a versatile gene that participates in a variety of developmental processes, particularly in chondrogenesis and male gonadogenesis. Campomelic dysplasia (CD), a severe developmental disorder characterized by systematic skeletal dysgenesis, has long been associated with SOX9 HI. However, the impact of SOX9 gene dosage on developmental processes has yet to be thoroughly investigated.

Many mouse models were used to explore the impact of SOX9 on bone growth. However, these models did not exhibit characteristics of CD in a manner analogous to humans, and no single model was able to replicate all associated phenotypes. HiPSCs possess the capacity to differentiate into various cell lineages, making our model suitable for studying the significance of SOX9 gene dosage during development. Since this is the first SOX9 HI model created in hiPSCs from a healthy donor to our knowledge, this allows us to research a wide range of developmental events and determine how the dose of the SOX9 gene affects development.

It has been reported that NMD targets one-third of the problematic mRNA as a cellular defense against mutation [46]. Given our selection of a splice site mutation for modeling CD, there is a possibility that NMD was activated in both HT and HM and degraded the mutSOX9 mRNA. In this particular SOX9 mutation, we did not find any alternation in the expression of NMD factors in the RNA-seq data. Furthermore, the RT-PCR showed that the HM and HT chondrocytes had successfully induced to express regions of SOX9 fragments, especially exon 1 to exon 2 when compared to their hiPSC counterparts. This suggests that mutSOX9 mRNA could be transcribed without triggering the NMD pathway with respect to WT.

Several experiments were carried out using our SOX9 HI hiPSC model to characterize these mutants in chondrocyte development, including alcian blue staining as a functional assay to examine the cell ability in accumulating GAGs as well as gene and protein expression. The amount of wild-type SOX9 dosage in hiPSC-derived chondrocytes varied according to their genotype both at the mRNA and protein levels. In addition, mutSOX9 hiPSC-derived chondrocytes showed diminished ability to accumulate GAGs. In IF staining, weak SOX9 protein expression could be observed in the cytoplasm of HM chondrocyte, suggesting that alternatively spliced mutSOX9 could still be transcribed and translated; however, the protein was not translocated or retained in the nucleus for transcriptional regulation. While weak SOX9 protein could be observed in the HM IF staining, SOX9 protein was absent in the Western blot result. Such a difference might be caused by the loss of epitope during protein denaturation of the Western blot analysis, yet the epitope was reserved in the IF staining and was recognized by the polyclonal primary antibody. Nonetheless, our models demonstrated significant differences in gene dosage between WT, HI, and HM systems at both RNA and protein levels, along with their resultant effects on chondrogenesis. In summary, HT chondrocytes exhibited reduced functionality compared to WT, reflecting the CD defects at a cellular level.

By using RNA-seq and RT-qPCR, the underlying pathways impacted by the SOX9 HI dosage were revealed. According to our findings, ribosome, BMP, ECM, and chromosome segregation related pathways were shown to be altered in SOX9 HI hiPSC-derived chondrocytes, which may help to clarify how skeletal dysplasia manifests in CD patients.

GSEA analysis revealed a statistically significant reduction in ECM and chromosomal segregation gene sets in mutant HI-derived chondrocytes. This finding is consistent with our knowledge about SOX9’s function during ECM remodeling. For example, Tsingas et al. demonstrated that SOX9 deletion led to intervertebral disc degeneration characterized by matrix remodeling [47]. SOX9 has been shown to positively regulate several ECM proteins in adult mice’s intervertebral discs [48]. These findings agree with the fact that SOX9 regulates chondrogenic ECM genes like COL2A1 and ACAN transcriptionally and promotes differentiation and homeostasis. A reduction of ECM gene expressions observed in the SOX9 HT model explained the underdeveloped and deformed cartilage tissues, such as tracheomalacia and clubfoot, in CD patients. While there is no literature reporting the relationship between SOX9 and chromosome segregation, research has shown that SOX9 upregulates chondrocyte proliferation rate [23], which may imply that SOX9 promotes cell proliferation by upregulating chromosome segregation processes. A reduction in chondrocyte proliferation rate produced by HI contributes to the smaller size of cartilages in CD, such as a flat nasal bridge and small chest.

The GSEA analysis revealed a significant upregulation in ribosomal gene expression. Ribosome biogenesis in chondrocyte maturation was associated with molecular link between rRNA transcription and transcriptional regulators of chondrocyte hypotrophy [49]. In mouse ATDC5 cells, Caron et al. found that genes related to ribosomes were elevated in SOX9i mutants [50]. The SOX9i mutants used in Caron’s study did not remove the SOX9 protein expression completely, which is similar to the SOX9 HI used in this study. Interestingly, the subsequent experiment showed a reduction in the protein translation capacity in the SOX9i model, despite an increase in ribosomal gene expression. Such an observation suggests SOX9’s role in chondrogenesis does not only involve in upregulating the matrix gene expression but also preparing the cell translational machinery to translate matrix genes. Chondrocytes require a robust machinery to maintain the ECM in the cartilage; a drop in SOX9 dosage weakens their ability to translate and retain the cartilage matrix, resulting in the underdeveloped skeletal system observed in CD.

Our HI hiPSC models derived from a healthy donor have successfully replicated the chondrocyte defects. The differentiated chondrocytes showed weaker ability in expressing COL2A1 and accumulating GAGs. GSEA analysis suggests alterations in ECM genes, chromosomal segregation, and ribosomal genes. These observations match with the current knowledge of SOX9’s role in regulating chondrocytes, revealing the pathogenesis of underdeveloped cartilage and bone development caused by SOX9 insufficiency in CD. Besides observing similar results from the previous research, the GO analysis revealed a bilateral interaction between the BMP-SMAD pathway and SOX9.

The relationship between SOX9 and BMP-SMAD signaling in chondrogenesis was thoroughly studied [51, 52]. SOX9 is generally considered as a downstream of the BMP signaling pathway. In this study, we were surprised to observe that the downstream transcriptome, such as ATOH8 and ID, and upstream BMP gene expression were both downregulated in the HI models. We can see that noggin is elevated in the HI mutant in the RT-qPCR validation. Noggin was generally reported to be inhibitory to chondrogenesis, as suggested in the experiments of C1 mesodermal stem cell line and the ATDC5 cell line in mice [53, 54]. However, its expression is also essential [55] to modulate BMP signaling for chondrocyte proliferation and inhibit premature hypertrophy in a temporal dependent manner [56–58]. Exogenous noggin even resulted in ectopic cartilage in trachea development and upregulation of SOX9 [59]. The idea of SOX9 regulating noggin was first proposed by Zehentner et al. in BMP2-induced chondrogenesis in mouse limb bud [60]. Zehentner showed that in BMP2-induced chondrogenesis, both SOX9 and noggin were upregulated. Disruption of the SOX9 protein would lead to noggin downregulation. The difference between BMP2 and BMP4 in chondrogenesis induction could be a reason for the opposite trend observed in noggin, as BMP2-induced chondrocytes showed a higher tendency toward hypertrophy [61].

Ohba et al. studied SOX9 transcriptional regulation using ChIP-sequencing in mice rib chondrocytes in 2015 [62]. They reported a list of SOX9 targets in supplementary file 1, and Noggin was one of the targets. SOX9 binds to its promoter region about 1k bp upstream to the transcription start site. They also included RNA-seq of overexpressing SOX9 in dermal fibroblast and attached the DEGs in supplementary file 3. Noggin was significantly, yet minutely, regulated with a log_2_ fold change at −0.6 in the SOX9 overexpression system. Their report bridged the noggin upregulation in the HT chondrocytes we observed in the BMP signaling pathway.

As the experiments from the above studies were performed in either rat or mouse chondrocytes, we performed ChIP-qPCR using our iPSC-derived chondrocyte models. With the assistance of the HOMER software, we shortlisted SOX9 predicted binding sites near the NOG regions. We found that at the NOG binding site 3, which is located 9 kb upstream of the NOG TSS, there was binding enrichment in both WT and HT chondrocytes. Also, WT showed a significant increase in enrichment compared to HT at this site. It is worth noting that within the ± 500 bp of binding site 3, the region has several predicted binding sites of SOX5, SOX6, and SOX9, according to the JASPAR CORE database. This suggests not only SOX9, but also the other two members of the SOX trio, SOX5 and SOX6, might bind to the distal regulatory region of NOG and modulate its expression during chondrogenesis. Our result suggested SOX9 plays a role in the BMP feedback mechanism via regulating NOG expression, and it is not just a downstream of BMP signaling.

According to earlier reports in the literature, HI genes could be rescued by replenishing gene expression. We investigated the potential for SOX9 overexpression upon dox induction during chondrocyte development to rescue SOX9 HI mutants. As evidenced by the gene expression and alcian blue staining results, our findings revealed that the rescue treatment was efficient in reversing not only the genotype but also the biochemical phenotype. Several therapeutic approaches have been proven effective for chondrogenesis in different disease settings via gene delivery [26], combined with our findings, this may pave the way for therapeutic approaches for diseases brought on by known HI genes, which were linked to numerous severe developmental abnormalities.

There were many extensive studies that elucidated the different facets of SOX9’s role in chondrocyte development using mice or rat samples. SOX9 is a downstream BMP and cooperated with SOX5 and SOX6 to promote chondrogenic genes such as COL2A1 and ACAN. SOX9 expression does not only regulate other gene expressions by binding to the genome; it can also interact with other regulators, such as RUNX2, and reduce their activities. SOX9 represses RUNX2 activity, either by direct interaction [63] or via BAPX1 expression [64], to prevent RUNX2 from further upregulating osteogenic genes. Such reports emphasize the importance of SOX9 dosage and its effects on chondrocyte development, its subsequent cell fate, and homeostasis. They explain the bowed bones, loss of a pair of ribs, and clubfoot observed in CD patients, who had insufficient SOX9 dosage to support the skeletal development due to a loss-of-function mutation in a SOX9 allele. In this study, we established a HI model, which demonstrated the chondrocyte defects observed in CD patients, from an hiPSC derived from a healthy donor. While we are not the first to report SOX9’s binding to NOG promoter regions, we confirmed such an interaction in a human model that SOX9 binds to a distal regulatory element of NOG and regulates its RNA transcription depending on the SOX9 dosage. We anticipate that the disrupted balance between noggin and BMP signaling identified in this study may potentially provide clues for the development of drugs, such as localized administration of noggin inhibitors, as a less intrusive treatment for CD and other chondrocyte abnormal disease patients.

## Conclusions

In our SOX9 HI hiPSC model generated from a healthy individual, we proved that the single point splice mutation in SOX9 reported in a CD patient could successfully lead to defected chondrocyte development in the hiPSC-derived chondrocytes with significant downregulation of the ECM gene set in the GSEA analysis, altered BMP and SMAD signaling pathways in the GO analysis, and weakened GAGs accumulation. The defected chondrocytes derived from the HI SOX9 model further confirm the causal relationship between SOX9 haploinsufficiency and skeletal deformities manifested in CD patients. Furthermore, our model can provide consensus observations from other reports regarding SOX9’s role during chondrocyte development.

We also found that the expression of BMP family inhibitor noggin was inversely correlated to the SOX9 dosage in our models, and this was further demonstrated by our ChIP-qPCR result showing that WT have more binding than HT at a distal regulatory elements site located at 9 kb upstream to the noggin TSS. These suggested that there is a feedback loop between BMP, SOX9, and noggin, in contrast to the common perception that SOX9 is one of the downstream genes in the BMP cascade during chondrogenesis. In our rescue models, we also found that overexpression of SOX9 could effectively restore COL2A1 expression and its ability to accumulate GAGs, implying the importance of SOX9 gene dosage.

With the help of our SOX9 HI hiPSC model, we hope to enhance the understanding of the significance of SOX9 gene dosage in relation to various developmental processes. This research could uncover additional mechanisms in other lineages, such as male gonadogenesis, potentially shedding light on how sex reversal and ambiguity manifest in CD patients.

## Supplementary Information

Below is the link to the electronic supplementary material.

Supplementary Methods (DOCX 17 KB)

Supplementary Figures (PDF 146 KB)

Supplementary Tables (XLSX 647 KB)

## Data Availability

The RNA-seq raw data, raw counts and normalized counts are available on Gene Expression Omnibus (GEO) at accession number: GSE253431.

## Abbreviations

CD: Campomelic dysplasia
CRIPSR: Clustered regularly interspaced short palindromic repeats
Cas9: CRISPR associated protein 9
CTCF: Corrected total cell fluorescence
DEGs: Differentially expressed genes
CM: Extracellular matrix
GO: Gene ontology
GSEA: Gene set enrichment analysis
hiPSCs: Human induced pluripotent stem cells
HI: Haploinsufficiency
HT: Heterozygous
HM: Homozygous
IF: Immunofluorescence
NMD: Nonsense-mediated decay
RCS: Rat chondrosarcoma
RT-qPCR: Reverse transcription-quantitative polymerase chain reaction
GAGs: Sulfated glycosaminoglycans
WT: Wild-type
