# Supplementary material for: SOX9 haploinsufficiency reveals SOX9-Noggin interaction in BMP-SMAD signaling pathway in chondrogenesis
Source: Cell Mol Life Sci. 2025 Mar 2;82(1):99. doi: 10.1007/s00018-025-05622-y (PMC11872873; doi:10.1007/s00018-025-05622-y)
Supplement: Supplementary file 1 — Supplementary Methods (DOCX 17 KB) [file 18_2025_5622_MOESM1_ESM.docx]

Supplementary Method

TA cloning

200 ng of genomic DNA from each clone and SOX9E2intron primers were used to amplify the insert in a 50 uL system by GoTaq® Master Mixes. 10 µL of PCR product was used in gel electrophoresis to confirm successful PCR, the rest of 40 µL was purified by the PCR Cleanup Kit (Geneaid, DFC100) according to the respective manual. pMDTH18-T Vector Cloning Kit (TaKaRa, 6011) was used. 1 µL of pMDTH18-T Vector, 0.3 pmol of insert DNA, and autoclaved water (Invitrogen, 10977015) were topped up to 5 µL and incubated at 16 °C for 30 min. The ligation product was transformed with 25 µL of E. coli DH5 α Competent Cells by the heat-shock procedure mentioned in the cloning of SOX9 sgRNA method. The recovered cells were spread on Salmon Gal (APOLLO SCIENTIFIC, BIMB1026) and IPTG (Sigma-Aldrich, I6758-1G) LB agar with 1 µg/ml of ampicillin and incubated at 37 °C for 16 h. White colonies were picked on next day in 10 µL autoclaved water, and 1 µL samples were used in colony PCR in a 30 uL system by GoTaq® Master Mixes. The rest of the PCR products were then sent out for Sanger sequencing after gel electrophoresis with a 10 uL sample.
