# Supplementary material for: SOX9 haploinsufficiency reveals SOX9-Noggin interaction in BMP-SMAD signaling pathway in chondrogenesis
Source: Cell Mol Life Sci. 2025 Mar 2;82(1):99. doi: 10.1007/s00018-025-05622-y (PMC11872873; doi:10.1007/s00018-025-05622-y)
Supplement: Supplementary file 2 — Supplementary Figures (PDF 146 KB) [file 18_2025_5622_MOESM2_ESM.pdf]

Supplementary. Figure 1

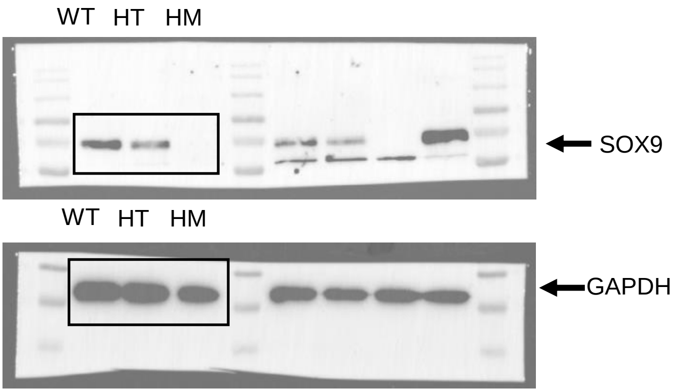

Supplementary Figure 1. Full-length blots of Western blot analysis of hiPSC-derived chondrocytes of Figure 3c.

Supplementary. Figure 2

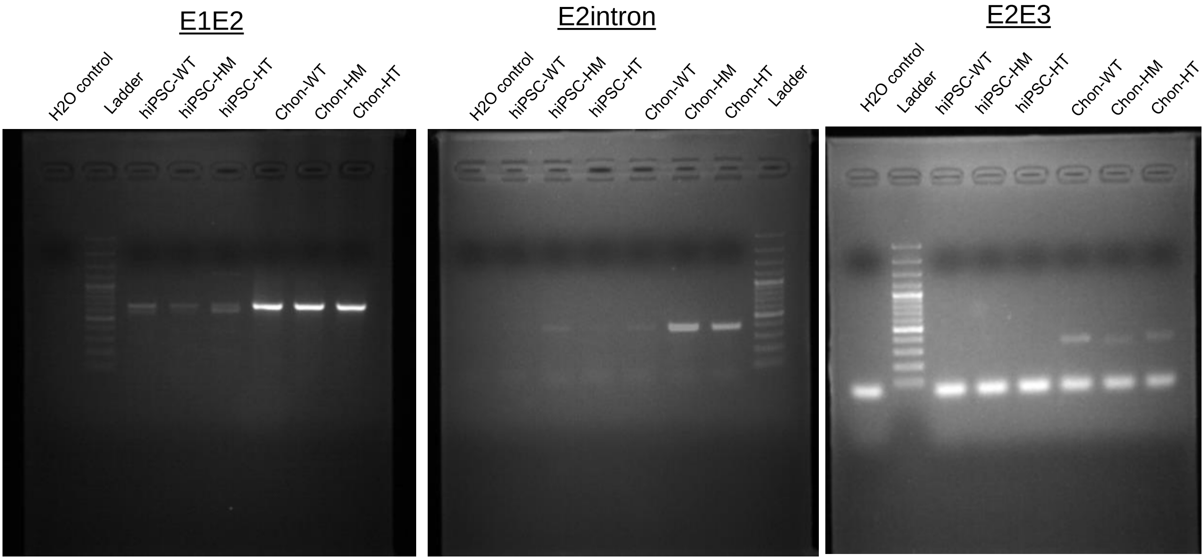

Supplementary Figure 2. Full-length gels of RT-PCR of hiPSC and hiPSC-derived chondrocytes (Chon) in Figure 4b. E1E2: Primers targeting exon 1 to exon 2 of SOX9 (mRNA: 653 bp; genome: 1549 bp); E2intron: Primers targeting exon2 to intron 2 of SOX9 (mRNA: 0 bp; genome: 392 bp). E2E3: Primers targeting exon 2 to exon 3 of SOX9 (mRNA: 348 bp; genome: 917 bp); Ladder: Thermo Scientific™ GeneRuler 100 bp Plus DNA Ladder (SM0322).
